# INDIVIDUAL CAREGIVING HISTORY TRAJECTORIES’ IMPACT ON LATER-LIFE SELF-PERCEPTIONS OF AGING

**DOI:** 10.1093/geroni/igae098.0866

**Published:** 2024-12-31

**Authors:** Rita Hu

**Affiliations:** University of Chicago, Chicago, Illinois, United States

## Abstract

This study assesses the impact of caregiving history trajectories and characteristics on later-life self-perceptions of aging (SPA) using data from the Health and Retirement Study. Caregiving history was collected using the event calendar method in the Life History Mail Survey, and SPA was measured in the 2016/2018 waves of the Psychosocial and Lifestyle Questionnaire. Employing sequence analysis and hierarchical cluster analysis on 2,215 participants, the research identifies caregiving history trajectories and examines their associations with later-life SPA, focusing on caregiving relationship types, duration, timing, and intensity. Four caregiving history trajectories were identified: Lifelong Diverse Caregivers, Later-life Spouse Caregivers, Parent Caregivers, and Later-life Non-Relative Caregivers. Linear regression analyses revealed that compared to caregivers in the Parent Caregivers cluster, those in the Lifelong Diverse Caregivers cluster were more likely to report higher negative later-life SPA (β= 0.18, SE = 0.06, p < 0.01) after controlling for current sociodemographic and health status. Also, earlier start age of caregiving, caring for child(ren), and caring for multiple people in the same year were all associated with higher later-life SPA. These associations remained significant after controlling for current sociodemographic characteristics. However, the associations were no longer statistically significant after adding in current health status. The findings reveal that caregiving experiences can be dynamic and lifelong and may have long-lasting impacts on later-life SPA. This highlights the necessity for targeted interventions to support caregivers’ well-being and suggests that current health status plays a crucial mediating role in the relationship between caregiving history and later-life SPA.

